# On the Cow-Pox

**Published:** 1814-10

**Authors:** Richard Walker

**Affiliations:** Oxford


*For the Medical and Physical Journal.*

*On the Cow-Pox;* by Mr. RICHARD WALKER, of Oxford.

*(Continued from p. 216.)*

*AUGUST 2d.*—I have just seen the observations of Mr. Rigby, a surgeon at Norwich, in the Medical Journal of this month, in which, in a concise and satisfactory manner, he removes a suggested difficulty or two respecting the propriety or necessity of using two punctures, &c. in vaccination. I am truly astonished at the various doubts and differences in opinion which have been occasionally started upon this subject, after the publication of the original facts by Dr. Jenner. Surely such persons must have been but little acquainted with the phenomena of inoculated small-pox, and the anomalous circumstances which sometimes, though rarely, occur in it. For my own part, I am thoroughly convinced that one puncture, according to the manner I have described above, is always sufficient. A multiplicity of punctures, indeed, may increase the chances of success in an ignorant or careless inoculator. Another difficulty, likewise, respecting the interference of the same modes in the proper progress of the disease, is equally ill-founded, and judiciously removed by the same gentleman.

Had I been the fortunate discoverer of vaccination, I should have been in great danger of losing the pecuniary reward it obtained, since, probably, I should have contented myself with barely stating the facts respecting it, as it appeared to me at the time of the discovery; and most probably, by the time the arbiters themselves had been satisfied respecting such facts, I should have stood in no need of it.

A correct decision, however, respecting the propriety of vaccine inoculation, is of such immense importance to the community, that however clear and unobjectionable it may have appeared to a great portion of the faculty, yet whilst another portion of the faculty, equally respectable, are throwing out apparent difficulties in the way of it, it can be no matter of surprise that so much caution and reserve has been adopted by those whose province it has been to decide between them.

The following brief statement of the ordinary progress of inoculated small-pox, which I drew up for my own use, some years ago, may not perhaps be unacceptable to others.

OBSERVATIONS.—*First.* From or on the 3d to 5th day inclusively, signs of infection having taken place.

*Second.* From 6th to 8th day inclusively, begin to sicken.

*Third.* From 7th to 9th, pustule in arm, fit to inoculate from; and sometimes so early as 6th day, and sometimes remains fit till 14th, or 19th, or longer.

*Fourth.* From 8th to 10th day, eruptions begin to appear.

*Fifth.* From 10th to 12th day, illness ceases.

*Sixth.* From 11th to 13th day, inflammation of arm at height.

*Seventh.* From 12th to 14th day, pustule from inoculation turned brown, but sometimes not till some days after.

*Eighth.* From 14th to 16th day, eruptions in face become ripe or maturated, and progressively so afterwards in other parts.

*Ninth.* By the 15th, or a few days after, the dried pustule in the arm begins to separate or loosen.

*Tenth.* By the 17th or 18th day, eruptions all turned; physicking may commence.

*Eleventh.* By the 24th or 25th day, physicking over, and fit to go airing a short time, previously to returning home.

N. B. In the estimation of days, the day of inoculation is included.

The above summary of the progress of inoculated small-pox was drawn up before the introduction of vaccination, and may still be useful or interesting.

*Cow-pox.*—The rules laid down for inoculated small-pox, may serve likewise for inoculated cow-pox; observing that, in general, the progress of this is commonly earlier by one day than that of the former, and the illness much slighter, scarcely claiming notice, and of shorter duration, and no eruption ordinarily; hence its termination is earlier, viz. by the 14th or 15th day usually.

On the 8th day, inclusively, from the time of inoculation, the pustule is sufficiently advanced to inoculate from ordinarily, and sometimes a day or two earlier.

The preparation for inoculated small-pox may commence from the time of inserting the variolous matter, by keeping the patients to a due regimen, giving three purgatives, viz. three doses of pulv. scammon. and calomel, between the time of inserting the matter and the time of sickening.

Vaccination requires no preparation either by regimen or medicine; and moreover is not liable to be taken by one person from another, as inoculated small-pox is.

The appearance and progress of the inoculated part in cow-pox is so like that in inoculated small-pox, although different in certain minute circumstances, clearly discernible to an accurate observer,* and so unlike any thing else we have ever seen in practice, as I should think must at

* The chief or characteristic difference between the pustule of the cow-pox and the small-pox is, that the fluid of the former changes at once from a serous state to dryness.

once impress every person, conversant in small-pox inoculation, with the close analogy, if not the identity, of one disease to the other; and consequently lead them to a very strong presumption, *à priori*, that vaccination might prove a safe substitute for it, or, in other words, a permanent preventive of small-pox, although such person were unacquainted with any previously-established fact respecting the security derived from cow-pox.

With respect to the opinion that certain diseases, as measles, chicken-pox, &c. coming subsequently to vaccination, may produce such a change in the system, as to render the person (unlike what happens in inoculated small-pox) susceptible of taking the small-pox; I can only say, that neither my experience nor reason accords with such a notion.

It has been said that noxious humours have been occasionally received into the habit, together with vaccine matter, producing noisome eruptions, &c. Such may adventitiously have happened; but nothing of this kind can be reasonably apprehended in transferring this matter, any more than in the instance of small-pox matter from one human subject to another.

Moreover, provided there be already some latent disease in the habit of the person inoculated, as scrophula, scurvy, ordinarily so called, &c. there is less danger of such disease being roused into action by vaccination, than by small-pox; and I presume it to be a universally admitted fact by the faculty, although supposed otherwise by ordinary persons, that neither scrophula nor scurvy can be communicated from one person to another by inoculation. In short, the only difficulty that ever presented itself to me respecting cow-pox, with regard to practice, was to distinguish between a pustule purely local in the part inoculated, in which the matter might not have been absorbed into the system, and the perfect one, in which the constitution had been affected; and, likewise, whether such local affection, insufficient to secure this patient from subsequent small-pox, be yet capable of communicating the cow-pox completely to a second person: that both these circumstances may happen, I have no doubt; but prudence dictates, that in the first instance the person be vaccinated afresh; and in the second, that matter under such dubious circumstances be not used. Each of these circumstances, we well know, may happen in inoculated small-pox;* and I am of opinion, that such a perplexing

* The matter of cow-pox being apparently of a milder or less virulent nature than that of small-pox.

circumstance is most securely obviated by attention to the mode of inoculation I have prescribed above, by which method the matter is not only perfectly inserted, but the part likewise excited to absorption.

We have had a few failures in Oxford, that is, attacks of small-pox after vaccination, particularly at the commencement of the practice; but probably they are all assignable to the causes above-mentioned; no such instance of failure has happened in my own practice; the circumstance of a local pustule is more likely to happen in the former than in the latter disease.

Much stress is laid, and very properly, on the circumstance that cow-pox is not contagious. Is inoculated small-pox contagious, when, as is sometimes though very rarely the case, it is free from eruptions, the disease venting itself by the pustule only at the inoculated part? Two cases of which I have mentioned here, and could enumerate an instance or two I have witnessed of the same kind, in all of which there was no subsequent small-pox.

With regard to any supposed dereliction or degeneracy in the matter or essence of cow-pox, in consequence of repeatedly passing through the human system, or imparting its specific efficacy to it, which might in time render it less active, and in the end inert, unless occasionally renovated from its original source, facts have not in the least induced me to give way to such an opinion, having lately vaccinated persons in whom the affection went on as actively and as satisfactorily as at the first introduction of the practice, although such affection had probably been propagated through an almost endless variety of persons without such renovation. I am therefore inclined to attribute the occasional variation in the apparent activity of the vaccinating matter, as in the variolous matter, not to any defect in the vaccinating matter itself, but to the peculiar idiosyncrasy of different habits, together with other adventitious circumstances. Moreover, if my *rationale* originally stated be true, viz. that the matter or essence of the vaccine disease possesses the power, or acts in assimilating to its own nature a certain portion of the fluids circulating in the system, and which in the end it expels, it must necessarily follow, that the essence or matter of this disease, as in the instance of small-pox, is unalterable in its specific nature, by passing over so repeatedly through the human system. It is not improbable, however, but that the matter, as taken from the cow, may, in being naturalized as it were to the human system, undergo a peculiar change or amelioration, which, when completed, it is no further susceptible tible of change or alteration, still retaining, nevertheless, its antivariolous efficacy as unimpaired as ever.

---

*On Specific Remedies.*

Nothing can be more pleasant or interesting to a novice in the profession of physic, than reading over the medical uses of a modern Herbal: every plant has its specific medical virtues, and every disease has its remedy. What a pity it is that progressive experience compels a practitioner to know otherwise, and, in the end, deprives him of a very great portion indeed of this faith! The Family Herbal of Dr. Thornton, incomparably superior to any work of the kind, unquestionably, which has preceded it, and far above the reach of my encomium or criticism, in other respects is, generally speaking, far too liberal or unguarded in its medical descriptions. It is proper that, in such a work, every plant should be retained that has any just claim to efficacy or utility, and such effects noticed; but, when we are told that certain plants which hold a subordinate place only in our present Pharmacopœias, and others which probably never had a place in any Pharmacopœia, possess specific virtues in curing the most formidable diseases, as horehound in consumption, misletoe in epilepsy,* &c. &c. it is

---

* Epilepsy occasionally ceases spontaneously, and thus has it happened that such a variety of medicines have obtained the credit of curing it.

Amidst an immense number of cases of epilepsy, which have presented to my observation, I have never been able to discover that any one of these medicines, or remedies, so called, exhibited any specific effect upon the disease. How a practitioner may be deceived in this respect, I shall show by the following fact. A boy became a patient of the Radcliffe Infirmary for this complaint, for which he took the nitrate of silver, at that time a newly-proposed remedy. In about a month, the fits, which had occurred frequently before, ceased; after continuing the medicine about a fortnight or three weeks longer, and remaining free from the fits, he was directed by his physician to be discharged cured. I requested, however, that the boy, who lived at a distance, might be retained for a time. In a week or two, the fits returned in like manner as before;—the medicine, which had all along been persisted in, was continued, and increased in quantity, for a due time, without any beneficial effect, and he was discharged. Had this boy been discharged before, we should probably have heard no more of him, and it would have been in vain for me, had I been disposed to have attempted it, to have persuaded any one that he was not cured by the medicine. Upon is probable ignorant persons may be misled, and the injury to themselves, in consequence, very serious.

It is true, this gentleman produces authorities for what he mentions, and very frequently the language itself of the authors; but very many of these instances of cures said to have been performed, positively, in this work, have long since been justly discredited by every practitioner of experience, and ought to be consigned to oblivion.* If what is asserted in this and other books of the kind be true, why tolleth the knell so frequently for persons arrested in the mid-day of life by a fatal disease? It is the frequent repetition of disappointment respecting medical facts, (so called,) I presume, which has reduced this gentleman to his present state of professional scepticism.

*Oxford.* RICHARD WALKER.

---

Upon interrogating the boy afterwards, I learned that he had had similar interruptions to his fits before, whilst taking no medicine.

It requires a much greater degree of experience than ordinarily falls to the lot of an individual practitioner, to determine the effect due to a medicine alone, exclusively of what is attributable to nature, regimen, &c. in certain diseases. This can be ascertained only by a sufficient number of facts on one side of the question or the other, as completely to remove all doubt; such, I take it, I have experienced.

I have witnessed the exhibition of very long-persevering trials, in a great number of instances, of the various medicines proposed as remedies in this disease, and no doubt they have each an equal claim to the title.

Should, however, the fits cease or abate during the use of any medicine, this certainly is an encouragement to administer it again in similar cases, provided it be of an innocent nature, but this is certainly not the case with nitrate of silver.

Epilepsy, arising from or depending upon causes which are removeable, as worms, injuries to the head, suppressed humours, certain mental affections, &c. ceases of course when those causes are removed.

* Should it be questioned how any person can be deceived in a plain matter of fact, as it might be called, I shall answer, and I well know with truth, that in such instances of cure, the nature of the complaint has been misunderstood; and this, as I likewise well know, may happen to a person of reputed eminence in his profession. I occasionally hear, or read, of cancers being cured by a specific remedy or application, that is, without excision or destruction by caustic: I may not doubt that a cure has been effected; but my own ample experience in such cases, together with my communication with other experienced persons of the faculty, compel me to disbelieve that such case was cancer.

